# Psychological Competences Mediating the Adoption of Health Behaviors in Adults Through Internet, Social Media and Online Games: A Systematic Review

**DOI:** 10.3390/bs16030357

**Published:** 2026-03-03

**Authors:** Matteo Mazzucato, Micol Savastano, Antonio Iudici

**Affiliations:** 1 Institute of Psychology and Psychotherapy, 35100 Padua, Italy; m.mazzucato@scuolainterazionista.it; 2 Department of Philosophy, Sociology, Education and Applied Psychology, University of Padua, 35131 Padua, Italy

**Keywords:** psychological factors, health behavior, self-efficacy, social support, health literacy, digital media use

## Abstract

Digital technologies such as the Internet, social media, and online games have become integral to an adult’s everyday life, yet their implications for health-related behaviors remain the subject of ongoing debate. While existing research has extensively examined risks and benefits of digital media use, evidence focused specifically on adult populations and on the psychological processes supporting health-oriented engagement remains fragmented. This systematic review with narrative synthesis, conducted in accordance with PRISMA 2020 guidelines, examined peer-reviewed studies published between 2015 and 2025 involving adults (≥18 years). Searches across PubMed, Scopus, Web of Science, and PsycINFO identified 27 eligible studies addressing spontaneous use of the Internet, social media, or online games in relation to actual health behaviors. Across studies, a consistent pattern emerged in which self-efficacy, health literacy, motivation, risk perception, and perceived social support were associated with the adoption of health-related behaviors, particularly physical activity, preventive practices, healthy eating, and health information seeking. However, the literature was characterized by a predominance of cross-sectional designs, a strong geographical concentration in East Asian contexts, and a marked imbalance across digital environments, with social media and informational Internet use being far more frequently studied than online games. Overall, the findings suggest that digital technologies are neither inherently beneficial nor harmful for adult health; rather, their effects depend on users’ psychological competencies and modes of engagement. By synthesizing evidence across digital contexts, this review proposes a competence-oriented framework that helps explain how everyday digital media use may translate into health-promoting behaviors in adulthood, while also highlighting critical gaps that future longitudinal, cross-cultural, and gaming-focused research should address.

## 1. Introduction

The widespread diffusion of digital technologies has profoundly reshaped everyday life across the lifespan. This transformation has significant implications not only for individual health, but also for public health systems, health education, and policy-making. The Internet, social media, and online games have become primary tools for communication, entertainment, information seeking, and access to services, including healthcare (Kemp, 2023; Pew Research Center, 2024). International data indicate that digital media use has steadily increased among adults of all age groups over the last two decades, with particularly marked growth among middle-aged and older adults, who have shown the fastest relative increases in Internet adoption (OECD, 2025; Kemp, 2023; Pew Research Center, 2024).

Despite their pervasiveness, the impact of these technologies on psychological and physical health remains the subject of intense scientific debate. Existing literature, largely focused on children and adolescents, report heterogeneous findings, highlighting both potential risks and health-related benefits. On the one hand, problematic forms of digital use have been associated with depression, anxiety, sleep disturbances, impaired psychosocial functioning, and other clinically relevant outcomes (Keles et al., 2020; Shannon et al., 2022; Kuss & Griffiths, 2017).

On the other hand, a growing body of evidence suggests that digital tools may also support health promotion by enhancing access to health information, fostering social support, strengthening psychological resources, and increasing engagement in health-related behaviors (Granic et al., 2014; Merolli et al., 2013; Chou et al., 2009).

Taken together, these findings suggest that digital technologies are neither intrinsically harmful nor inherently beneficial. Rather, their effects depend on how they are used and by whom (Orben & Przybylski, 2019; Valkenburg et al., 2022). However, despite this emerging consensus, the scientific debate remains fragmented with respect to the conceptualization of problematic digital use. In particular, no shared definition clearly distinguishes maladaptive and clinically relevant patterns of use from forms of digital engagement that may instead support physical and mental health (Billieux et al., 2015; Kardefelt-Winther, 2017).

Moreover, while digital technologies are now widely used for social, recreational, and health-related purposes across the lifespan, most empirical research has focused on younger populations considered at higher risk. Evidence on problematic and health-relevant forms of digital engagement in adult populations remains comparatively limited (Lopez-Fernandez et al., 2023), constraining the understanding of how different ways of using digital tools may translate into positive or negative health outcomes across adulthood.

Given the relevance of this debate for the scientific community, it is therefore necessary to provide an updated state of the art on this topic focusing specifically on adult populations. Although Internet use, social media engagement, and online gaming are often examined as distinct research domains, this distinction is largely tool-centered and does not necessarily reflect how digital use operates in everyday life. From this perspective, the object of analysis is not the digital medium per se—whose features evolve rapidly over time—but rather the psychological factors that shape how adults engage with digital environments and how such engagement translates into health-related behaviors. Consequently, focusing on a single digital tool risks fragmenting the evidence and obscuring shared mechanisms that operate across digital contexts (Billieux et al., 2015; Kardefelt-Winther, 2017).

Considering the current need (1) to systematize and update the evidence on this topic, (2) to offer a meaningful contribution to the debate on the effects of new technologies—by exploring how the Internet, social media, and online games may serve as tools for the intentional pursuit of health, (3) to adopt a theoretical framework that allows for a user-centered rather than tool-centered approach, and that remains informative despite the continuous evolution of digital technologies, the present review addresses the following research questions:

(a) What evidence emerges from the most recent literature regarding psychological factors capable of mediating the adoption of health-related behaviors in adult populations?
(b) Which health-related behaviors can be identified in relation to the use of the Internet, social media, and online games?
(c) Given the current state of research and technological development, which online contexts—among the Internet, social media, and online games—have been studied from a health-oriented perspective, and with what results for adult populations?

## 2. Theoretical Framework

Coherently with international and European efforts to conceptualize digital engagement in terms of citizens’ competencies (Triollet et al., 2023), this study adopts a competence-oriented perspective, grounded in the interactionist theoretical framework (Blumer, 1969; Iudici & Neri, 2024; Mead, 1934; Salvini, 1998).

Within this framework, psychological factors are examined as competencies, conceptualized as interactive modalities that enable adults to interpret, regulate, and intentionally use digital technologies in ways that may support health-related behaviors (See Table 1).

From an interactionist standpoint, the potential health-related effects of digital technologies are not determined by the intrinsic characteristics of the tools, but by how individuals use them. Using digital technologies to support health does not depend on exposure to specific online platforms, but on individuals’ ability to intentionally employ psychological competencies—such as self-regulation, reflexivity, and relational skills—in ways that promote health-related goals. Accordingly, health is conceptualized not as a fixed state, but as a dynamic process emerging from adopted behaviors and from the ongoing interaction between individual, social, cultural, and environmental factors (Turchi et al., 2022a; Turchi & Della Torre, 2007; Iudici et al., 2021; Iudici & Renzi, 2015).

This perspective is conceived in line with the most advanced theories and principal models of health promotion (Iudici et al., 2020). Rather, it is conceptually aligned with several widely used theoretical approaches that emphasize individuals’ agency, dialogical and self-regulatory capacities in health-related behaviors (Iudici & Corsi, 2017; Iudici, 2014; Turchi et al., 2019, 2022b) such as the Health Belief Model (Janz & Becker, 1984; Rosenstock et al., 1988), the Social Cognitive Theory (Bandura, 2004), the Theory of Planned Behavior (Ajzen, 1991) or the Self-Determination Theory (Deci & Ryan, 1985).

By centering on individuals’ active, intentional, and context-dependent use of digital environments, the present framework offers a theoretical lens capable of integrating both risk-oriented and benefit-oriented evidence, explaining how the same digital technologies may produce divergent health outcomes depending on the psychological competencies enacted by users.

## 3. Materials and Methods

### 3.1. Protocol and Reporting Standards

This systematic narrative review was conducted in accordance with the PRISMA 2020 guidelines. The review protocol was not prospectively registered in public databases (e.g., PROSPERO); however, the search, selection, and data extraction procedures were defined a priori and reported in accordance with the PRISMA 2020 guidelines. Although the protocol was not prospectively registered, all review procedures (search strategy, eligibility criteria, screening, and data extraction) were pre-defined, and any deviations were documented to ensure methodological transparency.

### 3.2. Eligibility Criteria

The eligibility criteria were structured according to a framework inspired by the PICo model (Population, Interest, Context), in which the “Interest” component was defined as the set of psychological factors relevant to the research, while the “Context” component was specified as the digital usage context (internet, social media, and video games). This framework was integrated with a specification of Outcomes, in order to include only studies that assessed the role of psychological factors in promoting the adoption of health behaviors.

#### 3.2.1. Population

Only studies conducted on adults (≥18 years) were included. Studies focused exclusively on children or adolescents were excluded. Studies with mixed-age samples were included only when adult data were clearly distinguishable or reconstructible.

Both general adult populations and adults living with chronic somatic conditions (e.g., diabetes, cardiovascular diseases, cancer survivorship) were eligible, provided that the study focus was on everyday digital use and psychological factors related to health behaviors, rather than on clinical treatment outcomes.

Studies conducted in exclusively clinical or psychiatric populations (e.g., individuals with diagnosed mental disorders, inpatients, or samples recruited within acute care or therapeutic settings) were excluded.

#### 3.2.2. Interest

Studies had to include at least one psychological factor, including self-efficacy, motivation, emotional regulation, coping, attitude, resilience, perceived social support, digital/health literacy, risk perception, and critical evaluation of information. To answer the research question, studies had to show which factors mediated, moderated, or explained the relationship between the use of internet/social media/video games and a health behavior.

#### 3.2.3. Context

Studies analyzing spontaneous use of internet, social media, or video games were included, i.e., not determined by an intervention, manipulation, or instruction from the researcher. Studies in which digital was an integral part of the intervention (web-based, eHealth, mHealth) or when it was used exclusively for recruitment or questionnaire administration were excluded.

#### 3.2.4. Outcomes

Selected studies had to report the development, adoption, or implementation of health behaviors such as physical activity, sedentary behavior reduction, healthy eating habits, adequate sleep, stress management strategies, adherence to medical treatments and participation in preventive practices, adoption of protective behaviors and risk avoidance (such as smoking abstinence or moderate alcohol consumption), as well as behaviors for maintaining social relationships. Studies reporting only intentions, attitudes, or beliefs (e.g., intention to exercise, vaccination intention) towards the hypothetical adoption of health behaviors were excluded. For research purposes, “information seeking” was considered a health behavior as it favored the acquisition of useful information for one’s health.

#### 3.2.5. Study Scope and Methodological Validity

The included studies primarily aimed to examine associations and, in some cases, mediation pathways between digital use contexts, psychological factors, and health-related behaviors in adult populations. Overall, the scope of the empirical evidence focused on identifying psychological factors through which everyday engagement with digital technologies may support or hinder health-related behaviors, rather than on evaluating the effectiveness of specific interventions.

The methodological validity of the included studies was supported by the use of validated psychometric instruments, study designs consistent with the stated research objectives, and analytic strategies aligned with the examined constructs. As reported in Table 1, most studies relied on established measures to assess psychological factors and adopted designs appropriate to their research questions, thereby providing a coherent and methodologically sound basis for the narrative synthesis.

Given the heterogeneity of study designs, outcomes, and measures, as well as the primary focus on identifying psychological processes rather than estimating pooled effect sizes, a formal quantitative risk-of-bias assessment was not conducted. Instead, methodological rigor was evaluated qualitatively through careful examination of study design characteristics, measurement validity, and consistency between objectives and analytic approaches.

### 3.3. Study Design

Empirical quantitative, qualitative, or mixed-methods studies were included, comprising cross-sectional, longitudinal, cohort, case–control, experimental, or quasi-experimental designs. Theoretical studies, narrative reviews, scoping reviews, meta-analyses, protocols without data, and contributions without original results were excluded.

### 3.4. Publication Features

Only articles published in peer-reviewed scientific journals and available in English were considered. The time window was limited to the period 2015–2025, a choice motivated by the significant evolution that digital tools and behaviors in the online context have undergone in the last decade, making more recent studies more relevant to the subject of this review. No geographical restrictions were applied. Studies conducted in any country were eligible for inclusion, provided that they met the other inclusion criteria.

### 3.5. Exclusion Criteria

Studies published before 2015 and non-peer-reviewed literature (e.g., theses, institutional reports, conference proceedings, editorials, opinion pieces) were excluded. Studies conducted on populations not relevant to the objective of this review, such as individuals under 18 years of age, animal studies, or laboratory-based research without human participants, were excluded. Studies conducted in exclusively clinical or psychiatric settings were excluded, including samples composed of patients with diagnosed mental disorders or individuals receiving structured medical or psychological treatment. Conversely, studies involving adults with chronic somatic conditions were included when the focus was on spontaneous digital use and psychological factors associated with health-related behaviors in daily life. Research based on structured digital interventions (e.g., web-based programs, eHealth or mHealth interventions), where digital use was prescribed or manipulated by the researcher, was also excluded.

### 3.6. Information Sources

The systematic search was conducted on the databases: PubMed, Scopus, Web of Science, and PsycINFO, selected for their complementarity.

### 3.7. Search Strategy

Search strategies were adapted to each database, using free keywords and controlled vocabularies (MeSH, APA Thesaurus). The searches were based on four conceptual blocks: (a) Digital usage contexts (internet, social media, video games); (b) health behaviors; (c) psychological factors; (d) adult population. When available, filters for human participants were applied to increase the accuracy of the search results.

Here is an example of a string used for searching the PubMed database:

(“self-efficacy”[tiab] OR “self efficacy”[tiab] OR motivation[tiab] OR “emotion regulation”[tiab] OR coping[tiab] OR resilience[tiab] OR “perceived social support”[tiab] OR “health literacy”[tiab] OR “digital literacy”[tiab] OR “risk perception”[tiab])

AND

(“physical activity”[tiab] OR exercis*[tiab] OR “healthy eating”[tiab] OR “diet quality”[tiab] OR “sleep quality”[tiab] OR “sleep hygiene”[tiab] OR “stress management”[tiab] OR “stress coping”[tiab] OR “support seeking”[tiab] OR “health information seeking”[tiab] OR “preventive behavior”[tiab])

AND

(“internet use”[tiab] OR “social media”[tiab] OR “social network*”[tiab] OR Facebook[tiab] OR Instagram[tiab] OR TikTok[tiab] OR Twitter[tiab] OR YouTube[tiab] OR “online communit*”[tiab] OR “online forum*”[tiab] OR “online gaming”[tiab] OR “video game*”[tiab])

AND

(mediat*[tiab] OR moderat*[tiab] OR predict*[tiab] OR associat*[tiab])

NOT

(“intervention”[tiab] OR “web-based”[tiab] OR “web based”[tiab] OR “eHealth”[tiab] OR “mHealth”[tiab] OR app[tiab] OR “mobile app”[tiab] OR “smartphone app”[tiab] OR randomized[tiab] OR randomised[tiab] OR trial[tiab] OR “RCT”[tiab])

AND

(“2015/01/01”[dp] : “2025/12/31”[dp])

AND

english[la]

NOT

(Review[pt] OR Meta-Analysis[pt] OR Editorial[pt] OR Comment[pt] OR Letter[pt])

NOT

(child[MeSH] OR adolescent [MeSH])

### 3.8. Study Selection

Screening occurred in two phases by two independent reviewers: (1) screening of titles and abstracts; (2) full-text review. Conflicts were resolved through discussion or through a third reviewer. All exclusions in the full-text phase were recorded.

**Figure 1:**
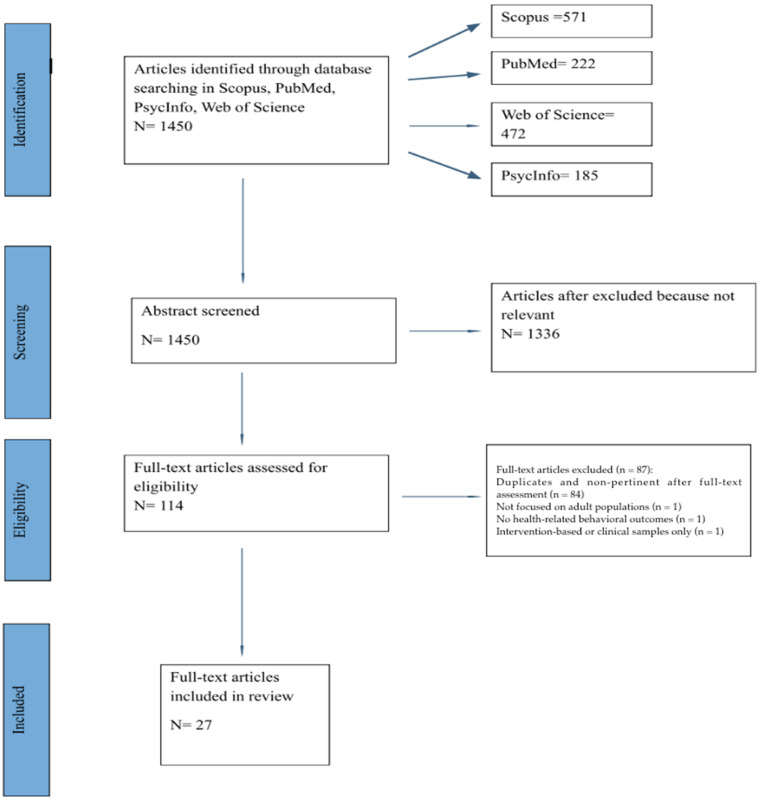
PRISMA 2020 Flow Diagram Process.

The process is illustrated below via PRISMA 2020 Flow Diagram (See Figure 1).

### 3.9. Data Extraction

For each included study, data were systematically extracted using a predefined and structured extraction grid, operationalized through the study characteristics table (Table 1). Extracted information included study design, sample characteristics, digital context, study objectives, psychological factors examined, health behaviors assessed, and the role attributed to psychological factors (i.e., predictor, mediator, or moderator).

A systematic narrative synthesis was conducted following an iterative and transparent procedure. Psychological factors were first identified at the individual study level and subsequently grouped into higher-order categories based on conceptual similarity and recurrence across studies. This process led to the identification of five core psychological competences consistently emerging across digital contexts.

To reduce the risk of author-driven bias, data extraction and categorization were independently performed by two reviewers, with discrepancies resolved through discussion until consensus was reached.

The included studies were characterized by clearly defined objectives, the use of validated psychometric instruments, and study designs consistent with their research aims, supporting the methodological validity of the evidence synthesized (See Table 2).

### 3.10. Data Synthesis

A systematic narrative synthesis was conducted to answer the research question, summarizing the psychological factors associated with the adoption of health behaviors through the use of the internet, social media, and online games. A meta-analysis was not feasible due to heterogeneity in psychological constructs, measurement tools, and outcome definitions across studies.

To synthesize the results, an attempt was made to use the same descriptive categories to facilitate a reading of what emerged (e.g., in the “physical activity” category, studies were included where, thanks to the use of digital tools, physical activity was initiated, maintained, or the frequency of physical exercise was increased).

## 4. Results

Across the 27 included studies, the evidence converges on a limited set of recurrent psychological factors mediating the relationship between everyday digital media use and adult health behaviors. Rather than platform-specific effects, the results indicate that health-related outcomes are primarily shaped by cross-cutting psychological competencies—self-efficacy, critical health literacy, risk perception, perceived social support, and motivation—that operate across digital contexts, although the available evidence is predominantly derived from social media-based studies.

Despite substantial heterogeneity in samples, measures, and outcomes, consistent patterns emerged for specific domains of health behavior. The most robust and recurrent associations concerned physical activity and preventive behaviors, while dietary behaviors and health information seeking showed more variable and context-dependent effects. Overall, the findings support a competence-oriented interpretation of digital engagement, whereby digital environments act as amplifiers or enablers of psychological processes rather than as direct determinants of health outcomes.

Given the marked heterogeneity in study design, methodological strength was explicitly considered in the synthesis of results. While the majority of included studies adopted cross-sectional survey designs, a smaller subset of field-based and analytically advanced studies (e.g., mediation and moderated mediation models) provided comparatively stronger support for hypothesized psychological pathways, although causal inference remains limited.

Evidence concerning self-efficacy and motivation was supported by field experiments and analytically robust mediation models, allowing more robust inferences regarding behavioral maintenance and change. In contrast, findings related to risk perception and health information seeking relied predominantly on cross-sectional designs, limiting causal interpretation. Qualitative studies were used to contextualize psychological mechanisms and user experiences but were not weighted equivalently to quantitative evidence when drawing conclusions.

To enhance conceptual clarity, outcomes were distinguished between enabling health behaviors and enacted health behaviors. Health information seeking was conceptualized as an enabling behavior, facilitating access to knowledge and supporting subsequent decision-making, rather than as a health outcome per se. In contrast, enacted health behaviors included physical activity, dietary practices, and preventive actions requiring sustained self-regulation and behavioral execution.

The synthesis indicates that enabling behaviors were primarily associated with competencies such as health literacy and risk perception, whereas enacted behaviors consistently required additional competencies—particularly self-efficacy, motivation, and perceived social support—to translate digital engagement into concrete and sustained health actions.

Regarding digital contexts, the evidence base was heavily skewed toward social media use, which accounted for the large majority of included studies. General Internet use as an informational environment was less frequently examined, while online video games were markedly underrepresented, with only one study directly addressing gaming-related health behaviors. This imbalance limits the generalizability of findings across digital environments and should be considered when interpreting cross-contextual conclusions.

In line with the adopted theoretical framework, the psychological factors identified in the included studies are interpreted here as competencies insofar as they are enacted through everyday digital use in ways that orient behavior toward health-related outcomes.

### 4.1. Self-Efficacy Competence

Across digital contexts, self-efficacy emerged as the most consistently associated psychological competence supporting enacted health behaviors, particularly physical activity and preventive practices. Cross-sectional evidence indicates that social media exposure and sharing of health-related content are associated with higher exercise-specific self-efficacy, which in turn relates to greater engagement in physical activity (Kashian & Liu, 2020; S. Liu & Miao, 2024).

Stronger methodological support for self-efficacy-related mechanisms was provided by field-based experimental evidence with longitudinal data in the dietary domain, showing self-efficacy processes linked to healthy eating maintenance in the context of eating-related social media posting (Kilb et al., 2023).

Self-efficacy was also examined in older adult populations, where Internet use was associated with physical activity through chain mediation models including self-efficacy and health literacy; however, these studies were cross-sectional and therefore do not allow causal inference (Liao et al., 2025).

Finally, in preventive behavior studies conducted during COVID-19, self-efficacy was repeatedly associated with protective behaviors, typically alongside risk perception and social norms, although the evidence was predominantly cross-sectional (Mahmood et al., 2021; Choi & Noh, 2023).

### 4.2. Critical Health Literacy Competence

Health literacy was primarily associated with enabling health behaviors, particularly online health information seeking and self-care practices. Across studies, higher levels of digital and health literacy were associated with indicators of improved information handling, reduced health-related uncertainty, and greater integration of online content into health-related decision-making (Van Duong et al., 2021; Mitsutake et al., 2023; Soroya et al., 2024).

The impact of health literacy on enacted behaviors such as physical activity and diet appeared more indirect and context-dependent, with evidence from chain mediation models suggesting an interplay with self-efficacy (Liao et al., 2025). Methodologically, evidence on health literacy relied almost exclusively on cross-sectional designs, limiting causal inference regarding behavior change processes.

### 4.3. The Competence to Anticipate, Recognize, and Manage Risks

Risk perception emerged as a key psychological mechanism primarily in studies examining preventive behaviors during infectious disease outbreaks. Across these studies, social media exposure was associated with heightened perceived susceptibility and severity, which in turn related to greater adoption of preventive behaviors (Choi, 2021; Oh et al., 2021; H. Liu, 2022; Tu & Li, 2024).

Outside emergency-focused outcomes, risk perception also appeared relevant to online health information seeking and related preventive practices. In particular, risk-related concerns were linked to Internet-based health information seeking (Lagoe & Atkin, 2015), and risk perception was integrated with social support processes in social media contexts relevant to preventive practices and health information seeking (Zhao et al., 2022).

Overall, the available evidence indicates that risk perception functions mainly as an attentional and motivational trigger whose association with health behavior is highly context-dependent; however, given the predominance of cross-sectional designs, the temporal ordering of these pathways cannot be established.

### 4.4. The Competence to Create Situations of Social Support

Perceived social support consistently facilitated both enabling and enacted health behaviors by fostering encouragement, accountability, and normative reinforcement within digital environments. Evidence converged on its relevance for physical activity and dietary maintenance, particularly in community-based or peer-oriented online contexts (Ling et al., 2015; Wheatley et al., 2021; Kilb et al., 2023).

While most quantitative findings were derived from cross-sectional designs, qualitative evidence provided convergent support by illustrating how emotional, informational, and relational support mechanisms sustain motivation and behavioral continuity over time (Wheatley et al., 2021).

### 4.5. Motivational Competence

Motivation, particularly autonomous forms of motivation, was consistently associated with physical activity outcomes across digital contexts. Evidence from observational, longitudinal, and qualitative studies suggests that digital platforms support sustained engagement when they align with users’ intrinsic goals, enjoyment, and sense of purpose (Zhou & Krishnan, 2019; Marquet et al., 2017; Bell et al., 2025).

Motivational processes appeared to interact closely with self-efficacy and perceived social support, reinforcing behavioral persistence rather than initiation alone. In contrast, externally regulated or controlled forms of motivation showed weaker and less stable associations with sustained health behaviors.

### 4.6. Contextual Specificity of COVID-19-Related Evidence

A substantial proportion of studies examining risk perception and preventive behaviors were conducted during the COVID-19 pandemic. These studies reflect a highly atypical digital context characterized by elevated emotional arousal, intensive institutional communication, heightened exposure to threat-related information, and constrained offline alternatives (Choi, 2021; Oh et al., 2021; H. Liu, 2022).

Compared with pre-pandemic studies, COVID-19 research showed a stronger and more immediate association between digital exposure, risk perception, and preventive behaviors. In contrast, pre-COVID evidence tended to report more gradual and cognitively mediated pathways linking digital use to health-related decision-making (Lagoe & Atkin, 2015; Zhao et al., 2022). Post-COVID evidence remains limited, preventing firm conclusions regarding the persistence of these mechanisms outside emergency contexts.

Accordingly, findings derived from pandemic-related studies were interpreted as context-specific and were analytically distinguished from pre- and post-COVID evidence to avoid overgeneralization to everyday digital health engagement.

## 5. Discussion

The results of this review allow us to address the initial research question concerning which psychological factors and competencies support the adoption of health behaviors in digital contexts. Across the included studies, five central psychological factors emerged consistently—self-efficacy, health literacy, risk perception, perceived social support, and motivation—through which it was possible to delineate a set of operational competencies relevant to health behavior adoption. Overall, the evidence suggests that these psychological factors contribute to a range of health-related behaviors, particularly physical activity, dietary choices, implementation of preventive practices, and health information seeking.

An important aspect that warrants specific consideration concerns the geographical distribution of the included studies. Although the evidence base includes research conducted in Western countries such as the United States and the United Kingdom, a substantial proportion of the studies were carried out in East Asian contexts, particularly China, South Korea, Japan, and Taiwan. This uneven distribution reflects the current state of the literature rather than a selection bias of the present review and should be taken into account when interpreting the findings. This pattern may partly reflect regional differences in digital adoption, platform ecosystems, and public engagement with digital health tools, as well as a strong research focus on the psychological and behavioral implications of digital media use in these contexts. At the same time, it raises questions regarding the extent to which the identified psychological factors operate similarly across cultural settings.

With respect to the digital contexts examined, the selected studies explored health-related behaviors in environments such as social media platforms, the Internet—primarily conceptualized as an informational space—and, to a more limited extent, video games. While these contexts collectively contribute to outlining a coherent psychological framework through which adults may engage in health-oriented behaviors, the evidence base is clearly skewed toward social media and informational Internet use. Studies explicitly focused on online games remain relatively scarce, a fact that calls for caution when generalizing the findings to health-related behaviors in the use of all technologies.

The prominence of self-efficacy across studies confirms and extends existing theories on its central role in behavioral change and health (Bandura, 2004). Evidence from studies such as Kashian and Liu (2020) and S. Liu and Miao (2024) expands upon earlier findings by Maher et al. (2014), who highlighted the effectiveness of structured social media-based interventions in enhancing physical activity-related self-efficacy. The present review suggests that even voluntary and unstructured engagement with digital platforms, including spontaneous content sharing and peer interaction, may strengthen self-efficacy, thereby broadening the potential applications of these mechanisms in health promotion.

Health literacy also emerged as a key factor supporting health behaviors in digital environments. While previous frameworks have emphasized technical skills related to online information searching (Norman & Skinner, 2006), the studies reviewed here highlight the crucial role of critical evaluation, interpretation, and application of health information (Nutbeam, 2000). In particular, findings from Van Duong et al. (2021) and Soroya et al. (2024) point to the importance of higher-order cognitive competencies, suggesting that digital health literacy should be conceptualized as a multidimensional construct rather than a purely technical skill set.

Risk perception emerged as a particularly relevant factor in contexts involving health threats and emergencies. This finding aligns with classic theoretical models such as Protection Motivation Theory (Rogers, 1975). However, studies such as Oh et al. (2021) and Choi (2021) extend prior work on the role of social media in shaping vaccine-related risk perceptions (e.g., Betsch et al., 2012) by showing that digital platforms can also positively amplify risk awareness, thereby promoting protective behaviors rather than merely facilitating misinformation.

Perceived social support further confirms its established role in health promotion (Holt-Lunstad et al., 2010). The reviewed studies, particularly Wheatley et al. (2021) and Ling et al. (2015), extend previous evidence on online social support (Zhang et al., 2020) by highlighting the importance of emotional support, mutual encouragement, and shared experiences within digital communities. These findings suggest that digital environments can foster active and relational forms of support that contribute meaningfully to health behavior adoption.

Motivational processes also emerged as relevant psychological competencies, consistent with self-determination theory (Ryan & Deci, 2020). Evidence from Zhou and Krishnan (2019) and Bell et al. (2025) integrates and extends previous findings on autonomous motivation in health behaviors (Teixeira et al., 2012), suggesting that digital environments may effectively support intrinsic and self-endorsed forms of motivation, thereby opening new possibilities for digital health interventions.

Taken together, these findings have important practical implications for health promotion interventions targeting adult populations in digitally mediated contexts. To foster health behavior adoption, interventions should aim to enhance users’ self-efficacy, promote critical and applied forms of digital health literacy, support accurate risk perception, facilitate access to online social support networks, and design digital experiences that nurture autonomous motivation. These competencies may serve as operational foundations for evidence-based interventions addressing both general health promotion and the prevention of problematic digital use.

Despite the value of the present findings, several limitations should be acknowledged. Most included studies relied on cross-sectional designs, limiting causal inference and the ability to capture dynamic changes over time. The predominance of studies conducted in East Asian contexts may further limit the generalizability of the findings to other cultural settings. In addition, heterogeneity in study designs and measurement approaches necessitated a narrative synthesis and precluded quantitative meta-analysis.

Future research should prioritize longitudinal and experimental designs to clarify causal pathways linking psychological factors, digital engagement, and health outcomes. Comparative cross-cultural studies and research focused on underrepresented digital contexts, particularly video games, are needed to address current gaps in the literature. Moreover, emerging technologies such as augmented reality and conversational artificial intelligence warrant investigation as potential tools for supporting health-related competencies and behaviors (Orrù et al., 2024).

## 6. Conclusions

The analysis of psychological factors and competencies that influence the adoption of health behaviors through digital tools reveals a complex and multifaceted landscape with substantial potential. The findings of this systematic review provide a useful conceptual foundation for rethinking health promotion in the digital era, with relevant implications for practice, future research, and health policy, while acknowledging the current limitations of the evidence base.

With respect to practice, the results suggest the importance of adopting a multidimensional approach when designing digital health interventions for adult populations. The reviewed evidence indicates that features aimed at supporting users’ self-efficacy—such as personalized feedback, graduated goals, and opportunities for mastery—may play a relevant role, alongside tools designed to enhance digital health literacy. In particular, supporting individuals’ ability to critically evaluate and apply online health information appears crucial in digitally mediated health contexts. Risk communication through social media also emerges as a sensitive domain, requiring a careful balance between effectiveness and responsibility in order to avoid both underestimation and excessive alarmism. In this regard, interactive data visualization techniques and tailored messaging strategies may represent promising avenues, although their effectiveness requires further empirical validation.

Moreover, the findings highlight the potential relevance of fostering safe and supportive online communities, which may complement or extend traditional forms of social support in digital environments. Similarly, the integration of gamification elements within digital health interventions appears as a promising opportunity, particularly in relation to motivation and engagement (Odgers & Jensen, 2020). However, such approaches should be implemented with careful ethical consideration, avoiding manipulative mechanisms and instead supporting autonomous forms of motivation consistent with users’ health goals.

Looking to future research, several priority directions emerge from the present review. Longitudinal and experimental studies are needed to clarify how the identified psychological factors—self-efficacy, health literacy, risk perception, perceived social support, and motivation—influence health behaviors over time and across different stages of adulthood. Further research examining the interactions among these factors may help to uncover synergistic or compensatory mechanisms that shape health outcomes in digital contexts. In addition, the personalization of digital interventions, informed by individual psychological characteristics and guided by transparent and ethically grounded algorithms, represents a promising but still underexplored research direction (Naslund et al., 2016).

As digital technologies continue to evolve, future studies should also examine the implications of emerging tools, such as artificial intelligence-based systems and immersive technologies, for health promotion. Importantly, such investigations should not only consider potential benefits, but also critically address ethical challenges related to privacy, autonomy, and equity in access to digital health resources.

The policy implications of these findings are noteworthy. The reviewed evidence underscores the need for guidelines and standards ensuring that digital health applications and platforms are grounded in psychological theory and empirical research, and that they adhere to established ethical principles. In parallel, training pathways for healthcare professionals may benefit from incorporating competencies related to digital health promotion and to the psychological mechanisms that underlie effective and problematic digital engagement (Smailhodzic et al., 2016). Public policies may further support research and innovation aimed at developing evidence-informed digital interventions that leverage psychological competencies to improve population health.

Overall, this review opens new perspectives for a more nuanced and user-centered approach to digital health promotion in adulthood. Translating this knowledge into effective, ethical, and accessible interventions remains a key challenge and will require sustained interdisciplinary collaboration among researchers, developers, healthcare professionals, and policymakers. An integrated and evidence-based approach is essential to ensure that digital health interventions are not only technologically advanced, but also psychologically informed, ethically sound, and equitably accessible, thereby supporting meaningful and sustainable improvements in public health outcomes.

## Figures and Tables

**Table 1:** Psychological factor and competence.

	Psychological Factor	Competence
Flow	Psychological Factor → Application in order to intentionally achieve a goal → Competence (knowing how to be and to do)	
Definition	Psychological Factor refers to an internal psychological attribute of the individual, encompassing relatively stable traits, temporary states, or acquired forms of psychological knowledge, that influence how individuals perceive, interpret, and respond to situations, thereby shaping their thoughts, emotions, motivations, and behaviors.	Competence refers to the intentional use and application of learned ways of acting—encompassing knowing how to act, how to relate, and how to position oneself—that enable individuals to interpret situations, anticipate possible developments, and deliberately decide how to act in order to achieve declared objectives across contexts.
Focus	State	Application
Example	Motivation	Use of motivation in order to achieve one’s goal thought contexts

**Table 2:** Detailed Characteristics of Included Studies.

Author and Year	Population	Psychological Factor	Context	Outcome (Health Behavior)	Study Objective	Study Design
(Lagoe & Atkin, 2015)	Adults, USA	Risk perception, health anxiety (validated scales)	Internet	Preventive practices	To examine how risk perception predicts online health information seeking and preventive practices	Cross-sectional survey
(Ling et al., 2015)	Users of online weight control communities	Perceived social support (validated social support scales)	Social Media	Healthy eating habits; physical activity	To assess the role of perceived social support in predicting health behaviors	Cross-sectional survey
(Deng & Liu, 2017)	Users of mobile social media health websites	Risk perception; perceived social support (validated scales)	Social Media	Preventive practices	To test the role of risk perception and social support in health information seeking	Cross-sectional survey
(Marquet et al., 2017)	College students, USA	Motivation (validated motivation questionnaires)	Videogames	Physical activity	To examine how motivations to play Pokémon GO relate to physical activity	Cross-sectional observational study
(Zhou & Krishnan, 2019)	Adults, USA	Motivation (validated psychographic measures)	Social Media	Physical activity	To investigate motivational predictors of exercise maintenance	Cross-sectional survey
(Zhang et al., 2020)	Users of WeChat	Perceived social support (validated scales)	Social Media	Social relationship maintenance	To model the pathway linking social support and health information seeking to well-being	Cross-sectional survey
(Kashian & Liu, 2020)	User of social media	Self-efficacy (validated self-efficacy scales)	Social Media	Physical activity	To assess whether sharing exercise content enhances self-efficacy and well-being	Cross-sectional survey
(Van Duong et al., 2021)	Adults, Taiwan	Digital/health literacy (validated literacy scales)	Internet	Healthy eating; physical activity; risk avoidance	To examine the association between digital health literacy and health behaviors	Cross-sectional survey
(Mahmood et al., 2021)	Adults, Pakistan	Self-efficacy; risk perception (validated scales)	Social Media	Protective behaviors	To test self-efficacy and perceived threat as mediators between social media use and preventive behaviors	Cross-sectional survey
(Choi, 2021)	Adults, South Korea	Risk perception; emotional response (validated measures)	Social Media	Preventive behaviors	To examine the effect of social media on risk perception and preventive behaviors	Cross-sectional survey
(Wheatley et al., 2021)	Adults, UK	Motivation; perceived social support (validated qualitative framework)	Social Media	Physical activity	To explore motivational and social support mechanisms in a physical activity campaign	Qualitative study
(Oh et al., 2021)	Adults, South Korea	Risk perception; emotional variables (validated scales)	Social Media	Preventive behaviors	To test risk perception as a mediator between social media use and preventive behaviors	Cross-sectional survey
(H. Liu, 2022)	Adults, China	Risk perception (validated scales)	Social Media	Preventive behaviors	To examine the impact of official social media on risk perception and public behaviors	Cross-sectional survey
(Ren et al., 2022)	Adults, China	Risk perception; self-efficacy (validated scales)	Social Media	Preventive behaviors	To assess the mediating role of risk and self-efficacy perceptions	Cross-sectional survey
(Zhao et al., 2022)	Users of social media	Risk perception; perceived social support (validated scales)	Social Media	Preventive practices	To integrate risk perception and social support in health information seeking	Cross-sectional survey
(Rupp & McCoy, 2023)	Adults, USA	Coping strategies (validated coping scales)	Social Media	Physical activity	To examine coping processes associated with body positivity and health behaviors	Cross-sectional survey
(Kilb et al., 2023)	Users of social media	Self-efficacy (validated self-efficacy measures)	Social Media	Healthy eating	To test self-efficacy mechanisms in eating-related social media posting	Field experiments with longitudinal data
(Choi & Noh, 2023)	Adults, South Korea	Self-efficacy (validated scales)	Social Media	Preventive behaviors	To assess self-efficacy as a mediator between social media exposure and preventive behaviors	Cross-sectional survey
(Mitsutake et al., 2023)	Adults, Japan	Digital/health literacy (validated scales)	Internet	Preventive practices	To examine health literacy in online health information seeking	Cross-sectional survey
(S. Liu & Miao, 2024)	Adults	Self-efficacy (validated exercise-related scales)	Social Media	Physical activity	To test self-efficacy as mediator between exposure to fitness content and physical activity	Cross-sectional survey
(Kukreti et al., 2024)	Users of social media	Risk perception; stress (validated scales)	Social Media	Preventive behaviors	To examine fear and risk perception predicting preventive behaviors	Cross-sectional survey
(Soroya et al., 2024)	Adults, Pakistan	Health literacy; health anxiety (validated scales)	Internet	Self-care behaviors	To assess health literacy and anxiety in predicting self-care behaviors	Cross-sectional survey
(Tu & Li, 2024)	Adults, China	Risk perception (validated scales)	Social Media	Protective and collective behaviors	To distinguish personal and societal risk perception in collective behaviors	Cross-sectional survey
(Bell et al., 2025)	Adults, UK	Motivation (validated SDT-based measures)	Social Media	Physical activity	To examine autonomous motivation as predictor of exercise behavior	Cross-sectional survey
(Liao et al., 2025)	Older adults, China	Self-efficacy; health literacy (validated scales)	Internet	Physical activity	To test chain mediation of self-efficacy and health literacy	Cross-section
(J. Liu et al., 2025)	Older adults, China	Risk perception, health anxiety (validated scales)	Social Media	Physical activity	To examine how risk perception predicts online health information seeking and preventive practices	Cross-sectional survey
(Batu et al., 2025)	Adults, Turkey	Perceived social support (validated social support scales)	Social Media	Healthy eating	To assess the role of perceived social support in predicting health behaviors	Cross-sectional survey

## Data Availability

The dataset presented is available under request.
